# 

**Published:** 1911-08-12

**Authors:** 


### NEW SPECIES OF TSETSE FLIES.

Newstead, in the "Annals of Tropical Medicine and Parasitology," December 20, 1910, describes three new species of the genus *Glossina*. These he terms *Glossina submorsitans*, *G. brevipalpis*, and *G. fuscipes*. The first is nearly related to *Glossina morsitans*, but may be told from it by the genital armature, which is structurally distinct. The second comes into the *Glossina fusca* group, being easily recognised from it, however, by its shorter and stouter palpi, its generally paler colour, the somewhat indefinite thoracic markings, the morphological characters of the male armature, and also by the presence of a long bilateral row or band of squamose spines. The third, *G. fuscipes*, is somewhere near *G. palpalis*, differing, however, in size (much smaller), also by the uniformly infuscated or dusky legs and by its dusky grey thorax. Its size really resembles *G. lachinoides*, but it is a relatively stouter-built insect, and altogether is much more like a dwarfed specimen of *G. palpalis* with infuscated legs and a dusky thorax. The example here described was taken by Dr. Shircore in Uganda.

### A HANDBOOK ON THE BILL.

Mr. John Murray has recently published in the form of a sixpenny pamphlet a very readable and critical examination of the National Insurance Bill by Ernest Schuster, LL.D. Owing to the important changes which have been introduced into the Bill during its consideration by Parliament many of the author's criticisms now no longer apply, but a study of this reprinted address will be found a convenient and easy way of becoming acquainted with the complexities of the Bill.

### BUILDINGS CONTEMPLATED AND IN PROGRESS.

Aldershot.—The building of the children's wing at the Aldershot Hospital has started under the superintendence of Mr. J. Davidson, A.R.I.B.A., whose plans for the original building were selected in open competition from some three hundred designs. The extension will cost about £2,000, and is to be a King Edward memorial.

Bangor.—Mr. Frank Bellis, architect, Bangor, has submitted plans of a new workhouse infirmary to the Board of Guardians. They were unanimously adopted and ordered to be sent to the Local Government Board.

Bolton.—Mr. J. Ward, of Bolton, is architect for the extensions to the Townleys Hospitals. Quantities may be obtained from the architect on deposit of £5.

Bradford.—The designs submitted in the new infirmary competition have been on exhibition at Field House, Bradford.

Exeter.—For alterations and additions at the Workhouse Mr. R. M. Challice is architect. Of six tenders received the lowest is £658, the highest £750.

Forden.—The Asylum Committee of the Forden Board of Guardians, requiring a water-supply on the site of the proposed new Workhouse, employed Mr. Mullins, of Bath, to advise them. He, by the aid of a divining-rod, indicated the presence of water at several points at a depth of about 130 feet, and estimated to sink a well, erect a windmill pump and 20,000 gallon reservoir, at a cost of over £400. No charge is to be made against the Guardians if water is not found.

Frimley.—Messrs. H. R. and B. A. Poulter are architects for the extension of the Frimley Cottage Hospital to be called the Georve V. wing. The foundation-stone has recently been laid, and it is hoped the necessary funds will be forthcoming before the opening ceremony.

Haywards Heath.—Messrs. Wheeler and Godman, of Horsham, are architects for the Edward Memorial Eliot Cottage Hospital at Haywards Heath. The foundation-stone was laid on July 7. The necessary capital, £3,120, is all in hand except £500, which has yet to be raised.

Mansfield.—Messrs. Vallance and Westwick, of Mansfield, are architects for alterations and additions to provide children's quarters and offices at the Stockwell Gate Workhouse. Tenders have been received, the lowest, by Mr. R. Moore, amounts to £999; the highest of the nine submitted amounted to £1,268.

### JOTTINGS.

The new Hôpital de la Pitié, Paris, built to replace the old institution of the same name, has just opened its doors. It has cost eleven million francs, or, roughly, £440,000, and consists of thirty-two medical and surgical buildings.

The new Home for Nurses at the Philippine General Hospital in Manila is completed and now in occupation. The beautiful building provides accommodation for thirty-eight nurses, and cost $125,000.

One of the interesting features of the new Hahnemann Hospital, Chicago, will be an assembly-room which will seat three hundred persons. It is to be used for various purposes, such as a chapel, lecture room, etc. The basement is to be provided with a gymnasium and swimming-bath.

### MEDICAL APPOINTMENTS.

Mr. Geoffrey Jefferson, M.B., B.S., F.R.C.S., has been appointed Demonstrator in Anatomy at the Manchester University. Mr. Jefferson graduated at London University, and was house surgeon in the Manchester Royal Infirmary in 1909-10. This year he was appointed one of its anæsthetists.

The following appointments have been made at Manchester Royal Infirmary: To be honorary consulting physician, Dr. Graham Steell; to be honorary consulting surgeon, Mr. G. A. Wright, F.R.C.S. Dr. R. T. Williamson, senior honorary assistant physician, has been appointed honorary physician, and Mr. J. Howson Ray, F.R.C.S. Eng., senior honorary assistant surgeon, has been appointed honorary surgeon. The house physician is Mr. J. C. Brentnall, M.R.C.S., L.R.C.P., and Mr. Edward Moir, L.S.A. Lond., Mr. E. Falkner Hill, M.B., Ch.B. Vict., Mr. G. Jefferson, F.R.C.S., have been reappointed anæsthetists. Dr. A. Ramsbottom was reappointed an assistant director of the Clinical Laboratory.

### APPOINTMENTS VACANT.

Full particulars of these vacancies can be obtained on reference to our advertisement columns:

Canterbury Borough Asylum.—Assistant Medical Officer.
Glasgow Parish Council.—Two Junior Resident Male Assistants.
Glasgow Western Infirmary.—Lecturer on Clinical Methods.
Queen's Hospital for Children, Hackney Road.—House Surgeon.
Royal Albert Hospital, Devonport.—Assistant House Surgeon.
Royal Surrey County Hospital, Guildford.—House Surgeon, Assistant House Surgeon.

### GIFTS.

Mrs. Josephine Laird, of Birkenhead, has left £300 to the Birkenhead Borough Hospital.

The Drapers' Company has given twenty guineas to the Seaside Convalescent Hospital, Seaford, Sussex.

An anonymous donor has sent £500 as a Coronation gift to the East London Hospital for Children, Shadwell, E.

Mr. Alexander Breeds, of Hastings, retired auctioneer and estate agent, has bequeathed £25 to the Free Dispensary.

Miss Ellen Pressly, of Bournemouth, has left £300 each to the Andover Cottage Hospital and to University College Hospital.

Mr. William Livingstone Russell, of Bath, has left £300 each to the Bath Royal United Hospital and to the Combe Down Convalescent Home.

Mr. Thomas Lee, of Liverpool, senior partner in the firm of Lee and Nightingale, advertising agents, has left £50 to the Cancer and Skin Hospital, Liverpool.

Mr. Lucius Octavius Hutton, of Dundrum, County Dublin, chairman of the Great Northern Railway Company of Ireland, has left £200 to the Royal City of Dublin Hospital.

Mrs. Fanny Boddy, of Liverpool, has left £200 to the Home for Female Incurables, Liverpool, and £100 each to the Royal Southern Hospital, the Liverpool Northern Hospital, and the Royal Infirmary, Liverpool.

The Queen's Hospital for Children, Hackney Road, has received a further £300 from the Zunz Bequest towards the support of the Annie Zunz Ward and £100 from the trustees of Mrs. Ogilvie's Charities.

The Rev. Charles William Herbert Kenrick, vicar of Holy Trinity, Barnstaple, Devon, has left £100 to the All Saints' Convalescent Home at Eastbourne, and £100 to the North Devon Infirmary at Barnstaple.

Miss Ann Brown, of Tiverton, has bequeathed £200 to each of the Homes at Walton-on-Thames, Bexhill-on-Sea, and Broadstairs of the Metropolitan Convalescent Institution, for the general purposes of those homes, and £100 to the Tiverton Infirmary.

Mr. Edward Bowen, wine and spirit merchant, has bequeathed £50 to the Great Northern Hospital, £100 to the Sydenham Wesleyan Methodist Circuit, and £50 to the Home and Dispensary for Sick Children at Lower Sydenham.

Captain John Foster, of Whitby, retired master mariner and steamship owner, has left £500 to the Whitby Cottage Hospital, and £600 to the Whitby Seamen's Hospital, with a request that the income therefrom should be paid to the inmates weekly or monthly.

Mr. Edward Henry Liddell, of Rye, Sussex, has stated that he had power of appointment for charitable purposes within the diocese of Winchester of certain property left by his late wife Mrs. Anne Maria Liddell, and he therefore appointed these funds in equal shares to the Royal County Hospital, Winchester, and to five other institutions.

### TO HOSPITAL SECRETARIES AND OTHERS.

We invite contributions from any of our readers, and shall be glad to pay, according to length, for items of news for the institutional section (including appointments, resignations, gifts, etc.) which we may publish. All payments for manuscripts used are made as early as possible after the beginning of each quarter.

